# Prevalence and associated factors of intimate partner violence against pregnant women in urban areas of Japan: a cross-sectional study

**DOI:** 10.1186/s12889-023-16105-9

**Published:** 2023-06-17

**Authors:** Naoko Maruyama, Shigeko Horiuchi, Yaeko Kataoka

**Affiliations:** 1 Graduate School of Nursing Science, St. Luke’s International University, 10-1 Akashi-Cho, Chuo-Ku, Tokyo, 104-0044 Japan; 2 Research Institute, National Center for Child Health and Development, 2-10-1 Okura, Setagaya, Tokyo 157-8535 Japan

**Keywords:** Intimate partner violence, Pregnancy, Prevalence, Risk factor, Cross-sectional study, Perinatal care

## Abstract

**Background:**

Intimate partner violence (IPV) against pregnant women is associated with many negative maternal and fetal outcomes and is a common public health problem all over the world. However, the issue has not been fully explored in Japan. This study aimed to explore the prevalence and risk factors of IPV against pregnant women in urban areas of Japan.

**Methods:**

This study was a secondary data analysis of a cross-sectional survey that was conducted on women beyond 34 weeks’ gestation in five perinatal facilities in urban areas of Japan, from July to October 2015. The sample size was calculated to be 1230. The Violence Against Women Screen was used for IPV screening. Multiple logistic regression analysis was used to calculate the adjusted odds ratio (AOR) with 95% confidence interval (CI) for risks of IPV while adjusting for confounding factors.

**Results:**

Of the 1346 women who participated in this study, 180 (13.4%) were identified as experiencing IPV. Compared to those who did not experience IPV (*n* = 1166 (86.6%)), women experiencing IPV had higher odds of being single mothers (AOR = 4.8; 95%CI: 2.0, 11.2), having lower household income (< 3 million yen, AOR = 2.6; 95%CI: 1.4, 4.6; ≥ 3 million yen and < 6 million yen, AOR = 1.9; 95%CI: 1.2, 2.9), having junior high school education background (AOR = 2.3; 95%CI: 1.0, 5.3) and being multipara (AOR = 1.6; 95%CI: 1.1, 2.4).

**Conclusions:**

13.4%, or about one in seven women, experienced IPV while pregnant. This high proportion indicates the need for policy to address the issue of violence against pregnant women. There is an urgent need to build a system for the early detection of victims that offers appropriate support to prevent the recurrence of violence while encouraging victim recovery.

**Supplementary Information:**

The online version contains supplementary material available at 10.1186/s12889-023-16105-9.

## Background

Intimate partner violence is a serious public health problem that affects almost one-third (27%) of women aged 15–49 years around the world [1]. The term “intimate partner violence (IPV)” describes any act by an intimate partner or ex-partner who intentionally attempts to control the victim using physical, sexual and/or psychological violence [1]. Pregnancy is a particularly vulnerable period for women. The global prevalence rate of any type of IPV during pregnancy was 25.0% in 118 studies including 124,838 women, and the rates varied within and between continents; the highest in Africa (36.1%) and the lowest in Europe (5.1%) [2]. IPV during this time has an additional effect on pregnancy-related morbidity and neonatal health outcomes; for example, miscarriage, stillbirth, pre-term delivery, and low birth weight babies [1]. Furthermore, children born to mothers subjected to maternal IPV were more likely to have delayed linguistic or neurological development [3] and behavioral problems [4]. Further, abused women were more likely to abuse their children, through actions such as shaking or suffocation, at 4 months after birth in comparison with non-abused women [5]. The pregnancy period is an ideal opportunity to detect IPV victims and support victims due to regular contact with healthcare providers throughout prenatal checkups [6, 7]. Therefore, perinatal healthcare providers should play an important role in addressing IPV.

In Japan, one-in-four women aged > 20 years who have had a spouse have experienced IPV [8]. The elimination of all forms of violence against women is a priority issue that should be addressed as a national responsibility in the 5th Basic Plan for Gender Equality [9]. In perinatal care, Horiuchi et al. [7] have developed a guideline that provides practical guidance on the detection of and support for the protection and safety of women who are victims of IPV. Furthermore, the Japan Academy of Midwifery has presented evidence on IPV screening methods and treatment for victims in the Midwifery Care Guidelines [10]. These are available free of charge in the clinical practice guideline database of the Medical Information Distribution Service within the Japan Council for Quality Health Care [11]. However, a national survey conducted in 2016 found that only 6.9% of antenatal care facilities perform IPV screening [12].

In promoting response to IPV at perinatal facilities nationwide, it is also necessary to consider regional characteristics. Japan is experiencing a long-term declining birth rate, and this trend is particularly noticeable in urban areas [13]. This suggests that there are regional differences in childbirth and child-rearing environments. Several previous studies have reported that the prevalence rate of IPV among pregnant women in urban areas of Japan was 4.6% (26 out of 562 women) [14], 16.1% (86 out of 533 women) [15], and 31.4% (111 out of 357 women) [16]. The reason for such a large range of reported rates could not be explained by differences in the screening tools used, study locations, and sample characteristics alone. In other words, the prevalence rate of violence against pregnant women in urban areas of Japan is unclear.

Identification of risk factors is critically important for informing strategies and programs to respond to IPV [17]. Globally, young age, low socio-economic status or income, low education, separated or divorced marital status, pregnancy, exposure to child maltreatment, depression, substance use, acceptance of violence, and exposure to prior abuse have been identified as risk factors for experiencing IPV among women [17]. According to a systematic review of studies conducted in eight countries and regions excluding Japan [18], the most important risk factors for IPV in women were unplanned pregnancy and having parents with less than a high school education. Young, unmarried women have also been reported to be at greatest risk of experiencing IPV [18]. Similar risk factors for pregnant women have been identified. Abuse before pregnancy, lower education level, pregnancy being unintended by either the victim or the perpetrator, lower socioeconomic status, and being unmarried were found to be predictors of abuse during pregnancy [19]. On the other hand, previous studies from Japan have not sufficiently examined demographic data, while only multiparity [16, 20] and having a history of physical abuse [16] have been identified as risk factors for pregnant women. Thus, the delay in identifying the prevalence and risk factors for IPV in pregnant women in Japan may be a factor that has prevented progress in efforts against IPV in perinatal care.

This study was a secondary data analysis of cross-sectional survey data [21] using a self-administered questionnaire about the loneliness of pregnant women in urban areas and related factors including IPV. We aimed to explore the prevalence and risk factors of IPV against pregnant women in urban areas of Japan.

## Methods

### Data collection

Recruitment was carried out in the outpatient department of a perinatal medical center at one general hospital serving relatively high-risk pregnancies, three obstetrician–gynecologist hospitals and one maternity clinic focused on low-risk pregnancies in Tokyo and Kanagawa, urban areas of East Japan. All facilities did not routinely perform IPV screening during prenatal checkups. Eligible participants were pregnant women who were beyond 34 weeks’ gestation and could read and write in Japanese. A researcher (NM) or a research assistant (nursing post-graduate student) or nursing staff from the research facilities distributed the questionnaire to eligible women at their prenatal checkups. Since prenatal checkups are made by appointment, nursing staff used the reservation system to keep track of pregnant women who were scheduled to visit the hospital each day. At the research facility, doctors conducted prenatal checkups, and nursing staff assisting with checkups knew in advance which women needed follow-up attention for various reasons such as psychological instability. Such women in need of special medical attention were excluded from this study. All other women who received the questionnaire were asked to answer the questions relating to their pregnancy. Completed questionnaires were returned to a collection box placed in the outpatient department or by mail between July and October 2015.

The sample size was calculated based on the ratio of single mothers to pregnant women because single mothers were considered to experience a high level of loneliness, as found in a previous study [22]. The ratio was estimated to be 3.5% by a national population census [23] and a national survey on single-parent families [24]. Therefore, to include more than 30 single mothers, the required number of pregnant women with a questionnaire recovery rate of 70% was calculated to be 1230.

A total of 1675 eligible women received the questionnaire, and 1402 questionnaires were collected (collection rate: 83.7%). When the questionnaire was placed in the collection box or returned by post, we regarded this as participants providing consent for study participation.

### Questionnaires

The questionnaire obtained information about women’s demographic characteristics and the IPV screening instrument. Additional file 1 shows this in more detail.

### Women’s demographics

The following demographics were included: age, marital status, possibility of becoming a single mother after the current delivery, working status, annual household income, educational background, previous history of psychiatric disorders, parity, any previous miscarriages or stillbirths or interrupted pregnancy experiences, and hospital treatment experience during the current pregnancy.

### Intimate partner violence

The Violence Against Women Screen (VAWS) [25] is a tool used to identify the rate and degree of IPV during pregnancy. The tool referred to women’s experience of abuse during the 12 months preceding the interview. The VAWS includes seven items, indicating physical, sexual, and psychological abuse, and has a single-factor structure. Validity and reliability were established for pregnant Japanese women using the General Health Questionnaire (*r* = 0.30), Self-Esteem Scale (*r* = -0.26), and Cronbach’s α value (0.70) [25].

In this study, we used the short version of the VAWS [26] which included four items: one item for physical violence (pulling or pushing), one item for sexual violence (forcing to have sex), and two items for psychological violence (feeling of fear created by what one’s partner does or says, and hitting the wall or throwing things). Women were asked to provide the frequency of experience for each item according to a three-point Likert scale: often (2), sometimes (1), or never (0). The score range was 0 to 9 when the calculation was weighted to the item of physical violence: often (3), sometimes (2), or never (0). A score of 2 or above indicated an experience of IPV. The short version of the VAWS had a sensitivity of 100% and a specificity of 97.6% compared to the Index of Spouse Abuse tool [26], which is the gold standard for IPV screening tools.

The Cronbach’s α was 0.68 for the 1346 participants in the present study, indicating fairly good reliability. To confirm the one-dimensional nature of the scale, confirmatory factor analysis was conducted using Amos Graphics (Sonora, California, USA) [27]. We created a hypothetical model in which IPV was explained by four VAWS observed variables, and assessed it in combination with multiple model fit indices; the chi-square value (CMIN), GFI (Goodness of Fit Index), CFI (Comparative Fit Index), RMSEA (Root Mean Square Error of Approximation), and SRMR (Standardized Root Mean square Residual). This is because the chi-square value (CMIN) tends to be significant when the sample size is large, and GFI and CFI tend to show good values when the number of variables included in the model is small [28]. The criteria for each model fit indices were as follows: lower chi-square value is better, GFI and CFI are better than 0.9, RMSEA and SRMR are better than 0.05 and worse than 0.1.

As a result, the following model fitness indices were found: CMIN = 26.4(*p* = 0.000), GFI = 0.990, CFI = 0.972, RMSEA = 0.095, and SRMR = 0.0286. Although the CMIN value was large and significant, the GFI and CFI values were 0.9 or more, and the RMSEA value was close to 0.1, but the SRMR value was 0.05 or less. Therefore, it can be said that the fit of the model assuming that the short version of the VAWS is a single-factor structure was good. Thus, we decided to evaluate IPV using the total score of the short version of the VAWS.

### Ethical considerations

All study processes were conducted in accordance with the Declaration of Helsinki, and due consideration was given to protecting human rights. Voluntary participation, the right to refuse to answer without penalty, and survey respondent anonymity were assured, and these were explained through written information about the research, which was provided to each participant. Precautions were taken to protect the safety and privacy of participants, and the need to complete the questionnaire by the participant was emphasized on the front page of the questionnaire. If the study participants wanted information about resources such as IPV consultation centers and phone numbers, they could obtain this from outpatient research facilities. They were also informed that they could consult with nursing staff regarding any queries they had regarding the questionnaire. With the cooperation of the nursing staff, we refrained from distributing questionnaires to women who were likely to be burdened by answering the questionnaires. However, in the unlikely event that the participant experienced emotional distress as a result of answering the questionnaire, they could consult a certified social worker or a counselor at the expense of the researchers. However, no adverse events and no cases requiring counseling with a specialist were reported during the survey period.

### Data analysis

The dataset analyzed during this study is shown in Additional file 2. Basic statistics for the demographics and responses to the survey measurements were calculated and analyzed. The IPV prevalence rate was calculated by using the established cut-off point for the short version of the VAWS tool. Next, to clarify the relationships between each demographic variable and IPV, the *χ*^2^ test or Fisher’s exact test was conducted. Multiple logistic regression analysis was used to identify the risk factors for IPV. Although some variables were not found to be significantly associated with IPV, they were considered to be potentially important variables based on previous research. Therefore, all variables except“marital status”, which was strongly correlated with the variable “possibility of becoming a single mother after current delivery”, were entered into the logistic regression model, and analyzed using a forced entry method to adjust for confounding factors. A forced entry method is a direct approach and is best if there are no a priori-hypotheses about which variables have greater importance than others [29]. All analyses were conducted using SPSS for Windows, Version 28 J (SPSS Japan Inc.). All *P* values were two-sided; *P* < 0.05 was considered to indicate a statistically significant difference.

## Results

### Characteristics of participants

Overall, 1346 of the distributed 1675 questionnaires had no missing data from the VAWS tool; these data were then analyzed (response rate = 80.4%). The age range of participants was 17–46 years, and the mean age was 31.9 years (SD = 4.9). The mean number of pregnancy weeks was 35.7 weeks gestation (SD = 1.6) and 51.5% were primipara. Almost all participants (97.5%) were married, and nearly half of them (46.6%) were working.

### Prevalence of IPV

The total score range of the VAWS tool was 0 to 9 points, and the mean score was 0.57 points (SD = 1.2). The number of women positive for IPV screening was 180 (13.4%), which meant ≥ 2 points in the VAWS tool. For the psychological violence question, “Do you feel frightened by what he does or says?”, 136 women (75.6%) out of 180 responded “sometimes” and 12 women (6.7%) responded “often”. For another psychological violence question, “Has your partner hit the wall or thrown an object?”, 114 women (63.3%) responded “sometimes” and eight women (4.4%) responded “often”. For the sexual violence question, “Has your partner forced you to have sex?”, 86 women (47.8%) responded “sometimes” and 15 women (8.3%) responded “often”. For the physical violence question, “Has your partner pulled your arm, pushed, and/or slapped you?”, 63 women (35%) responded “sometimes” and six women (3.3%) responded “often”.

### Risk factors for IPV victims

Table 1 shows the association between IPV and each demographic variable. IPV was found to be associated with age, marital status, possibility of becoming a single mother after the current delivery, working status, annual household income, educational background, parity, and previous miscarriage or stillbirth or interrupted pregnancy experience.

**Table 1:** Comparison of demographic characteristics:women experiencing IPV vs women not experiencing IPV (*N* = 1346)

			**women experiencing IPV**		**women not experiencing IPV**			
	***n***	**(%)**	***n* = 180**	**(%)**	***n* = 1166**	**(%)**	*P-*value*	**Effect size**
Age (years)								
< 20 s(17–29)	397	(29.5)	65	(37.1)	332	(29.0)	0.03	.060
> 30 s(30–46)	924	(68.6)	110	(62.9)	814	(71.0)		
Missing	25	(1.9)	―^**^		―			
Marital status								
Married	1312	(97.5)	171	(96.1)	1141	(98.4)	0.07	.056
Unmarried	26	(1.9)	7	(3.9)	19	(1.6)		
Missing	8	(0.6)	―		―			
Possibility of becoming a single mother after the current delivery								
Yes	31	(2.3)	16	(9.0)	15	(1.3)	0.00	.174
No	1306	(97.0)	161	(91.0)	1145	(98.7)		
Missing	9	(0.7)	―		―			
Working status								
Working (full-time or part-time work)	627	(46.6)	66	(37.1)	561	(48.3)	0.01	.076
Non-working	712	(52.9)	112	(62.9)	600	(51.7)		
Missing	7	(0.5)	―		―			
Annual household income (millionYen)								
< 3	127	(9.4)	32	(18.6)	95	(8.4)	0.00	.165
3–6	588	(43.7)	95	(55.2)	493	(43.8)		
≥ 6	582	(43.2)	45	(26.2)	537	(47.7)		
Missing	49	(3.6)	―		―			
Educational background								
Junior high school	46	(3.4)	17	(9.4)	29	(2.5)	0.00	.174
High school	280	(20.8)	52	(28.9)	228	(19.6)		
Junior college or career college	459	(34.1)	64	(35.6)	395	(34.0)		
University or higher degree	558	(41.5)	47	(26.1)	511	(43.9)		
Missing	3	(0.2)	―		―			
Previous history of psychiatric disorder								
Yes	138	(10.3)	20	(11.1)	118	(10.1)	0.69	.011
No	1208	(89.7)	160	(88.9)	1048	(89.9)		
Parity								
Primipara	693	(51.5)	70	(39.5)	623	(53.7)	0.00	.096
Multipara	645	(47.9)	107	(60.5)	538	(46.3)		
Missing	8	(0.6)	―		―			
Previous miscarriage or stillbirth or interrupted pregnancy experience								
Yes	425	(31.6)	71	(39.4)	354	(30.4)	0.02	.066
No	919	(68.3)	109	(60.6)	810	(69.6)		
Missing	2	(0.1)	―		―			
Hospital treatment experience during current pregnancy								
Yes	43	(3.2)	8	(4.5)	35	(3.0)	0.26	.029
No	1295	(96.2)	169	(95.5)	1126	(97.0)		
Missing	8	(0.6)	―		―			

*Abbreviation:* *IPV* Intimate partner violence

^*^The *P*-values were calculated using the chi-squared test or Fisher's exact test

^**^ “-” in the table were missing data and were not included in the statistical test

Table 2 shows the odd ratios (ORs) and 95% confidence intervals (95%CI) from logistic regression analysis of IPV screening that adjusts for the women’s demographic variables described above. Four factors were found to be significantly associated with IPV. First was the possibility of becoming a single mother after the current delivery (AOR = 4.8; 95%CI: 2.0, 11.2), which meant that the women with the possibility of becoming single mothers after the current delivery were 4.8 times more likely to experience IPV than women without this possibility. The second factor was lower household income wherein the risk for IPV was higher among women with household earnings of < 3 million yen (AOR = 2.6; 95%CI: 1.4, 4.6) and ≥ 3 million yen and < 6 million yen (AOR = 1.9; 95%CI: 1.2, 2.9). This suggests that compared to women with a household income of 6 million yen or more, women with less than 3 million yen were 2.6 times more likely to experience IPV, and women with 3 million to less than 6 million yen were 1.9 times more likely to experience IPV. Third was having a highest educational background in junior high school (AOR = 2.3; 95%CI: 1.0, 5.3), which meant that women with a final education in junior high school were 2.3 times more likely to experience IPV than women with university or higher degrees. The fourth factor was being multiparous (AOR = 1.6; 95%CI: 1.1, 2.4), which meant that multipara was 1.6 times more likely to experience IPV than primiparas.

**Table 2:** Factors associated with IPV against pregnant women

	**Unadjusted OR**	**[95%Cl]**	**Adjusted OR**	**[95%Cl]**	*P*-value*
Age (years)					
< 20 s(17–29)	1.4	[1.0, 2.0]	1.3	[0.9, 1.9]	0.17
> 30 s(30–46)	1		1		
Possibility of becoming a single mother after the current delivery					
Yes	7.6	[3.7, 15.6]	4.8	[2.0, 11.2]	0.00
No	1		1		
Working status					
Working	1		1		0.43
Non-working	1.6	[1.1, 2.2]	1.2	[0.8, 1.7]	
Annual household income (million Yen)					
< 3	4.0	[2.4, 6.6]	2.6	[1.4, 4.6]	0.00
3–6	2.3	[1.6, 3.3]	1.9	[1.2, 2.9]	0.00
≥ 6	1		1		
Educational background					
Junior high school	6.4	[3.3, 12.4]	2.3	[1.0, 5.3]	0.04
High school	2.5	[1.6, 3.8]	1.3	[0.8, 2.1]	0.32
Junior college or career college	1.8	[1.2, 2.6]	1.2	[0.8, 1.8]	0.46
University or higher degree	1		1		
Previous history of psychiatric disorder					
Yes	1.1	[0.7, 1.8]	0.9	[0.5, 1.6]	0.70
No	1		1		
Parity					
Primipara	1		1		0.01
Multipara	1.8	[1.3, 2.4]	1.6	[1.1, 2.4]	
Previous miscarriage or stillbirth or interrupted pregnancy experience					
Yes	1.5	[1.1, 2.1]	1.3	[0.9, 1.9]	0.12
No	1		1		
Hospital treatment experience during current pregnancy					
Yes	1.5	[0.7, 3.3]	1.9	[0.8, 4.3]	0.15
No	1		1		

Total number of participants used in the analysis was 1276 (Women experiencing IPV: *n* = 168; Women not experiencing IPV: *n* = 1108)

The factor of "marital status" was strongly correlated with the factor "possibility of becoming a single mother after the current delivery"; therefore, only the factor "possibility of becoming a single mother after the current delivery" was used for multivariate logistic regression analysis

*Abbreviations*: *IPV* Intimate partner violence, *OR* Odds ratio, *CI* Confidence interval

^*^The *P*-values were calculated using logistic regression analysis

## Discussion

### Prevalence of IPV

We showed that around one in seven women (13.4%) experienced IPV while pregnant. In Japan, 25% of women aged > 20 years who have had a spouse have experienced violence from their spouse [8]. Thus, as in many other countries and settings [30], the results of this study showed a lower prevalence of IPV during pregnancy than the lifetime prevalence. The prevalence of women who experienced IPV in our study was lower compared to previous studies among pregnant women in urban areas of Japan [14–16]. However, our findings were similar to that of a study conducted among 79,222 pregnant women in another part of Japan [31]. Therefore, the prevalence of 13.4% in this study could be somewhat generalizable to urban pregnant women in Japan. This high prevalence indicates the need to promote efforts against IPV in perinatal care.

It is difficult for victims to disclose IPV in healthcare settings due to various barriers. Such barriers include healthcare providers having a negative attitude towards victims or their disclosure for example, by being unsympathetic, disinterested, and insensitive/inattentive to the victims' perceptions of safety and concerns about the consequences of disclosing abuse [32]. On the other hand, directly asking victims about violence in a safe and confidential environment encourage victims to disclose abuse [32] and these experiences are more beneficial than harmful for women, due to the therapeutic process of talking about the abuse [33].

In Japan, 41.6% of women who had experienced violence had not consulted anyone about it while only 1.9% of women had consulted a healthcare provider about abuse [8]. Therefore, it is essential to build a safe and supportive system and environment where perinatal healthcare providers implement IPV screening for all pregnant women and directly ask them about abuse.

### Risk factors for IPV victims

We identified four risk factors for IPV against pregnant women in this study. The first factor was the possibility of becoming a single mother after the current delivery. Women with the possibility of becoming single mothers after the current delivery included those who at the time of the interview were unmarried or divorced; or were going through a divorce. Marital discord and being unmarried have been reported as risk factors for IPV in previous studies [17, 20, 34], which is consistent with our results.

The second factor was having a lower annual household income of < 3 million yen and 3 million yen and < 6 million yen. In Japan, the average household income for a family with children was around 6 million yen ten years ago and has increased to around 8 million yen in recent years. The poverty line for the past ten years is around 1.2 million [35]. Therefore, the results of this study showed that the lower the household income, the higher the risk of IPV.

The third factor was having highest educational background in junior high school, which meant the women with a final education in junior high school were 2.3-folds more likely to experience IPV compared to women with a university or degree or higher. Our findings of the association between IPV against pregnant women and low-income households and low education were also consistent with previous studies [17, 20].

Finally, multiparous women were 1.6-fold more likely to experience IPV than primiparous women. The finding that multipara was more likely to experience IPV than primipara was consistent with previous Japanese studies [16, 20]. Globally, unintended or unplanned pregnancy is a known risk factor for IPV [18, 19]. Therefore, multipara should especially be assessed for unintended or unplanned pregnancy due to violence. Perinatal healthcare providers need to assess whether IPV victims have these risk factors and refer them to the support they need for independence and recovery from violence.

In Japan, the proportion of single-mother households among households with children is around 7.0% [35]. Although the number of households with children is decreasing, the number of single-mother households has not changed significantly, so this proportion is gradually increasing [35]. And the poverty rate of Japan's total population is 15.7%, which is particularly high among developed countries that are members of the OECD [36]. By household type, the poverty rate for two or more adult households with children is less than 10%, while, the poverty rate for single adult households with children is about 50% [37]. Comparing household income, the average household income of single-mother households is 3.7 million yen, which is significantly lower than the average household income of 8.1 million yen for all households with children [38]. Furthermore, the average household income of single-mother households whose mother's final educational background is junior high school is even lower at 2.7 million yen [38]. Thus, poverty among single mothers is a serious issue in Japan. This situation may make it difficult for women who are victims of IPV to leave their perpetrators or to become independent and recover from violence after they leave their perpetrators. Therefore, support for victims of violence should be considered along with other issues faced by single mothers in Japan. It is also noteworthy that the proportion of children in single-mother households is high in areas with low income and a high out-migration rate [39]. Therefore, it is important to consider the response to IPV in the context of urban areas.

### Clinical implications

Our study has several implications. First, it provided some insights for clinical implementation for IPV screening in perinatal care. This study demonstrated that as many as 1402 pregnant women answered questionnaires distributed at the outpatient department of the perinatal medical center in a general hospital, obstetrician–gynecologist hospitals, and maternity clinics, despite the voluntary nature of research participation. This means that by adding IPV screening items to the routine questionnaires administered in the outpatient department at various types of facilities, IPV screening can be performed for all pregnant women.

Second, this study provided evidence about the prevalence of IPV and the multiple risk factors of IPV. IPV is a pervasive issue for Japanese pregnant women and women experiencing IPV may require socioeconomic support. These results also suggest the importance of understanding the characteristics of IPV victims and the need for IPV education for healthcare providers. Moreover, some evidence shows that IPV training may improve healthcare providers’ attitudes towards IPV, and improve knowledge and self-perceived readiness to respond to those affected by IPV [40]. The e-learning about IPV for midwives and perinatal nurses developed in Japan was found to be effective in improving knowledge but did not result in behavioral change [41]. The best strategy for promoting the uptake and use of IPV evidence for practice has not been specified [42]. Hence, more effective education programs for perinatal staff need to be developed to promote understanding of IPV victims and implementation of screening and subsequent support.

### Limitations and further research

This present study has some limitations. First, the participants were recruited from pregnant women beyond 34 weeks’ gestation excluding those with preterm delivery. We also excluded women who potentially had mental health conditions as judged to require attention in follow-up visits by nurses at the healthcare facilities. Excluding these groups of women may have limited our findings to apparently healthy pregnant women with term pregnancy. Thus, the prevalence and risk factors we have identified may not be generalizable to pregnant women in the first and second trimesters of pregnancy or those with various health conditions.

Second, this was a cross-sectional descriptive study; therefore, the causal relationship between some variables and the occurrence of IPV could not be inferred.

The third limitation relates to the fact that this study is a secondary analysis of data collected in 2015. Globally, the coronavirus disease of 2019 (COVID-19) and ensuing pandemic further increased women’s exposure to violence, as a result of measures such as lockdowns and disruptions to vital support services [43]. Our results do not reflect the multiple effects of COVID-19; therefore, the prevalence we found may be lower than what it is in reality and some risk factors may have been exacerbated [44].

In the future, a longitudinal study that expands the study area and the health level of pregnant women is needed.

## Conclusion

This study was conducted to identify the prevalence and risk factors of IPV in urban areas of Japan. We found that around one in every seven women (13.4%) experienced IPV while pregnant. Women who had the possibility of becoming single mothers after the current delivery, those from low-income households, with a junior high school education background, and were multiparous were found to be at higher risk of IPV. These findings underscore the importance of building a system that detects victims early and offers appropriate support to prevent the recurrence of violence while encouraging victim recovery during the perinatal care period.

## Supplementary Information

**Additional file 1.** Questionnaire.

**Additional file 2.** The data of 1346 subjects used for analysis.

## Data Availability

The dataset is not publicly available. All data generated or analyzed during this study are included in this article and its additional files.

## Abbreviations

IPV: Intimate partner violence
AOR: Adjusted odds ratio
CI: Confidence interval
VAWS: The violence against women screen
CMIN: The chi-square value
GFI: Goodness of fit index
CFI: Comparative fit index
RMSEA: Root mean square error of approximation
SRMR: Standardized root mean square residual
SD: Standard deviation
ORs: Odd ratios
COVID-19: Coronavirus disease 2019

## References

[1] World Health Organization. Violence Against Women Prevalence Estimates, 2018: Global, regional and national prevalence estimates for intimate partner violence against women and global and regional prevalence estimates for non-partner sexual violence against women. Geneva: World Health Organization; 2021. https://www.who.int/publications/i/item/9789240022256. Accessed 6 Sept 2022.

[2] Romàn-Gàlvez RM, Martin-Pelàez S, Fernàndez-Félix BM, Zamora J, Khan KS, Bueno-Cavanillas A (2021). Worldwide prevalence of intimate partner violence in pregnancy. A systematic review and meta-analysis. Front Public Health.

[3] Udo IE, Sharps P, Bronner Y, Hossain MB (2016). Maternal intimate partner violence: relationships with language and neurological development of infants and toddlers. Matern Child Health J.

[4] Bianchi AL, McFarlane J, Cesario S, Symes L, Maddoux J (2016). Continued intimate partner violence during pregnancy and after birth and its effect on child functioning. J Obstet Gynecol Neonatal Nurs.

[5] Amemiya A, Fujiwara T (2016). Association between maternal intimate partner violence victimization during pregnancy and maternal abusive behavior towards infants at 4 months of age in Japan. Child Abuse Negl.

[6] World Health Organization. Responding to intimate partner violence and sexual violence against women. WHO clinical and policy guidelines. Geneva: World Health Organization; 2013. http://apps.who.int/iris/bitstream/10665/85240/1/9789241548595_eng.pdf. Accessed 6 Sept 2022. 24354041

[7] Horiuchi S, Yaju Y, Kataoka Y, Grace Eto H, Matsumoto N (2009). Development of an evidence-based domestic violence guideline: supporting perinatal women-centred care in Japan. Midwifery.

[8] Gender Equality Bureau, Cabinet Office of Japan. 2020 Survey on violence between men and women. https://www.gender.go.jp/policy/no_violence/e-vaw/chousa/h11_top.html. Accessed 19 Apr 2023.

[9] Gender Equality Bureau, Cabinet Office of Japan. Main Policies. https://www.gender.go.jp/english_contents/mge/violence/index.html. Accessed 19 Apr 2023.

[10] Guidelines Committee of the Japan Academy of Midwifery. 2020 Evidence-Based Guidelines for Midwifery Care. https://www.jyosan.jp/uploads/files/journal/210311-JJAM_2020Evidence-Based_Guidelines_Midwifery_Care_Final2.pdf. Accessed 19 Apr 2023.

[11] Minds Tokyo GRADE Center, Minds Guidelines library. https://minds.jcqhc.or.jp/english. Accessed 19 Apr 2023.

[12] Inoue S, Kataoka Y, Eto H (2020). A survey of care policies for low risk pregnancies among obstetrics institutions in Japan. J Jpn Acad Midwif.

[13] Ministry of Health Labour and Welfare of Japan. Live birth by prefecture. In: Specified Report of Vital Statistics in FY2021. https://www.mhlw.go.jp/english/database/db-hw/FY2021/live_births.html. Accessed 30 May 2023.

[14] Kita S, Haruna M, Matsuzaki M, Kamibeppu K (2016). Associations between intimate partner violence (IPV) during pregnancy, mother-to-infant bonding failure, and postnatal depressive symptoms. Arch Women’s Ment Health.

[15] Kita S, Tobe H, Umeshita K, Hayashi M, Kamibeppu K (2021). Impact of intimate partner violence and childhood maltreatment on maternal-infant maltreatment: a longitudinal study. Jpn J Nurs Sci.

[16] Inami E, Kataoka Y, Eto H, Horiuchi S (2010). Intimate partner violence against Japanese and non-Japanese women in Japan: a cross-sectional study in the perinatal setting. Jpn J Nurs Sci.

[17] World Health Organization. Preventing intimate partner and sexual violence against women: Taking action and generating evidence. Geneva: World Health Organization; 2010. http://whqlibdoc.who.int/publications/2010/9789241564007_eng.pdf. Accessed 6 Sept 2022.

[18] Yakubovich AR, Stöckl H, Murray J, Melendez-Torres GJ, Steinert JI, Glavin CEY (2018). Risk and protective factors for intimate partner violence against women: systematic review and meta-analyses of prospective-longitudinal studies. Am J Public Health.

[19] James L, Brody D, Hamilton Z (2013). Risk factors for domestic violence during pregnancy: a meta-analytic review. Violence Vict.

[20] Kita S, Kataoka Y, Porter SE (2014). Prevalence and risk factors of intimate partner violence among pregnant women in Japan. Health Care Women Int.

[21] Maruyama N. Pregnant women’s loneliness: correlated factors and its impacts on maternal role identification and common complaints during pregnancy. J Jpn Acad Midwif. 2017;31(1):23–33. 10.3418/jjam.31.23. (Abstract in English, Maintext in Japanese).

[22] Keating-Lefler R, Hudson DB, Campbell-Grossman C, Fleck MO, Westfall J (2004). Needs, concerns, and social support of single, low-income mothers. Issues in Mental Health Nurs.

[23] National Statistic Center. e-Stat Portal site of Official Statistics of Japan: 2010 Population Census. https://www.e-stat.go.jp/en/stat-search/files?page=1&layout=datalist&toukei=00200521&tstat=000001039448&cycle=0&tclass1=000001045009&tclass2=000001046265&tclass3val=0. Accessed 19 Apr 2023.

[24] Ministry of Health Labour and Welfare of Japan. 2011 Nationwide Survey on Single parent household. https://www.mhlw.go.jp/seisakunitsuite/bunya/kodomo/kodomo_kosodate/boshi-katei/boshi-setai_h23/. Japanese. Accessed 6 Sept 2022.

[25] Kataoka Y. Development of the violence against women screen. Jpn J Nurs Sci. 2005;25(3):51–60. 10.5630/jans1981.25.3_51. (Abstract in English, Maintext in Japanese).

[26] Imazeki M, Kataoka Y. Examination of accuracy of revised edition “Violence Against Women Screen. J Jpn Acad Midwif. 2013;26(3):122. (In Japanese).

[27] Alavi M, Visentin DC, Thapa DK, Hunt GE, Watson R, Cleary M. Exploratory factor analysis and principal component analysis in clinical studies: Which one should you use? [published online ahead of print, 2020 Apr 7]. J Adv Nurs. 2020. 10.1111/jan.14377. 10.1111/jan.14377 32255218

[28] Alavi M, Visentin DC, Thapa DK, Hunt GE, Watson R, Cleary M (2020). Chi-square for model fit in confirmatory factor analysis. J Adv Nurs.

[29] Stoltzfus JC (2011). Logistic regression: a brief primer. Acad Emerg Med.

[30] Devries KM, Kishor S, Johnson H, Stöckl H, Bacchus LJ, Garcia-Moreno C (2010). Intimate partner violence during pregnancy: analysis of prevalence data from 19 countries. Reprod Health Matters.

[31] Tanoue K, Nishigori H, Watanabe Z (2021). Interannual changes in the prevalence of intimate partner violence against pregnant women in miyagi prefecture after the great East Japan earthquake: the Japan environment and children's study. J Interpers Violence.

[32] Heron RL, Eisma MC (2021). Barriers and facilitators of disclosing domestic violence to the healthcare service: a systematic review of qualitative research. Health Soc Care Community.

[33] Snyder BL (2016). Women's experience of being interviewed about abuse: a qualitative systematic review. J Psychiatr Ment Health Nurs.

[34] Fazel S, Smith EN, Chang Z, Geddes JR (2018). Risk factors for interpersonal violence: an umbrella review of meta-analyses. Br J Psychiatry.

[35] Ministry of Health Labour and Welfare of Japan. 2019 Comprehensive Survey of Living Conditions. https://www.mhlw.go.jp/english/database/db-hss/cslc-report.html. Accessed 19 Apr 2023.

[36] OECD Data. Poverty rate. https://data.oecd.org/inequality/poverty-rate.htm. Accessed 19 Apr 2023.

[37] OECD Family Database. 4. Child outcomes: CO2.2 Child poverty. https://www.oecd.org/els/soc/CO_2_2_Child_Poverty.pdf. Accessed 19 Apr 2023.

[38] Ministry of Health Labour and Welfare of Japan. 2021 Nationwide Survey on Single parent household. https://www.mhlw.go.jp/stf/seisakunitsuite/bunya/0000188147_00013.html. Accessed 19 Apr 2023.

[39] Abe Y, Kawabata M, Shibatsuji Y (2021). Spatial clustering patterns of children in single-mother households in Japan. J Spat Econo.

[40] Kalra N, Hooker L, Reisenhofer S, Di Tanna GL, García-Moreno C (2021). Training healthcare providers to respond to intimate partner violence against women. Cochrane Database Syst Rev.

[41] Maruyama N, Kataoka Y, Horiuchi S (2022). Effects of e-learning on the support of midwives and nurses to perinatal women suffering from intimate partner violence: a randomized controlled trial. Jpn J Nurs Sci.

[42] MacGregor JCD, Wathen N, Kothari A, Hundal PK, Naimi A (2014). Strategies to promote uptake and use of intimate partner violence and child maltreatment knowledge: an integrative review. BMC Public Health.

[43] World Health Organization. Devastatingly pervasive: 1 in 3 women globally experience violence. Geneva, New York: World Health Organization; 2021 https://www.who.int/news/item/09-03-2021-devastatingly-pervasive-1-in-3-women-globally-experience-violence. Accessed 6 Sept 2022.

[44] Moreira DN, Pinto da Costa M (2020). The impact of the Covid-19 pandemic in the precipitation of intimate partner violence. Int J Law Psychiatry.

