# Supplementary material for: Prevalence and associated factors of intimate partner violence against pregnant women in urban areas of Japan: a cross-sectional study
Source: BMC Public Health. 2023 Jun 17;23:1168. doi: 10.1186/s12889-023-16105-9 (PMC10276381; doi:10.1186/s12889-023-16105-9)
Supplement: Supplementary file 1 — Additional file 1. Questionnaire. [file 12889_2023_16105_MOESM1_ESM.docx]

Questionnaire


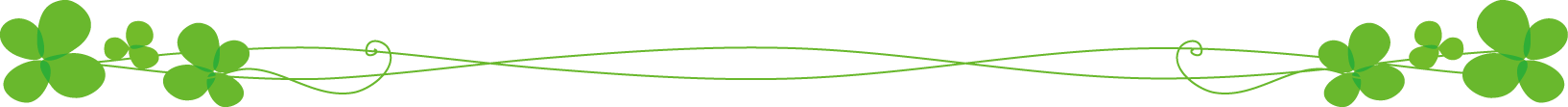


＊　Please answer **before giving birth.**

＊　Please answer alone without consulting with anyone.

＊　There are 6 questionnaire sheets on both sides and it takes about 10 minutes to

answer.

＊　After answering, please put it in the enclosed envelope and post it in the collection box of the questionnaire about feelings and support of pregnant women

installed on the hospital floor. (or please put it in the mailbox.)

Thank you for your cooperation.

　　　　　　　　　　　　　　　　　　　　　　　　　
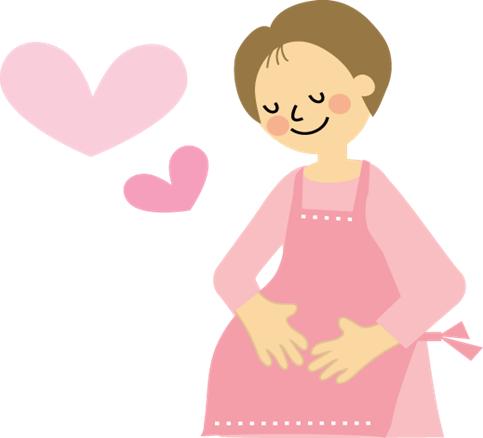


Note: Excerpts from only the items used in the analysis of this study.

【Question1】Please answer about your **current pregnancy.**

Please fill in the number in parenthesis and select an applicable answer.

| 1. How many weeks are you pregnant today? | （　　　　　）weeks | |
| --- | --- | --- |
| 2. How many times have you given birth this time?  (Excludes miscarriage and stillbirth) | １．First | ２．2nd or more |
| 3. Did you receive hospital treatment due to complications during pregnancy? | １．Yes | ２．No |

【Question2】　Finally, please answer about you.

Please fill in the number or specific matters in parentheses and select an applicable answer.

About question 2, please select all applicable answers.

| 1．How old are you? | （　　　　　）years old |
| --- | --- |
| 2．Are you legally married？ | １．Yes |
|  | ２．No, I plan to register our marriage. |
|  | ３．No, I have no plans to get married. |
| 3．Do you have the possibility of becoming a single mother  after the current delivery? | １．Yes |
|  | ２．No |
| 4．Do you have a job? | １．Yes |
|  | ２．No |
| 5．How much is your household income for the last year?  Household income is the income of the entire household, including the income of the family living together. | １．<3 million Yen |
|  | ２．3-6 million Yen |
|  | ３．>6 million Yen |

　　　　　　　　　　　　　　　　
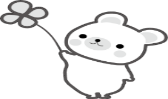


| 6．What is your educational background？ | １．Junior high school |
| --- | --- |
|  | ２．High school |
|  | ３．Junior college or career college |
|  | ４．University or higher degree |
| 7． Questions 9.-12. ★ are questions about your relationship with your partner over the past year.  ★Do you feel frightened by what he does or said? | １．Never |
|  | ２．Sometimes |
|  | ３．Often |
| 8.★Has your partner hit the wall or thrown an object? | １．Never |
|  | ２．Sometimes |
|  | ３．Often |
| 9.★Has your partner forced you to have sex? | １．Never |
|  | ２．Sometimes |
|  | ３．Often |
| 10.★Has your partner pulled your arm, pushed, and/or slapped  you? | １．Never |
|  | ２．Sometimes |
|  | ３．Often |
| 11．Have you ever had a miscarriage, stillbirth or abortion? | １．Yes |
|  | ２．No |
| 12．Have you been treated for a mental illness? | １．Yes |
|  | ２．No |

　　
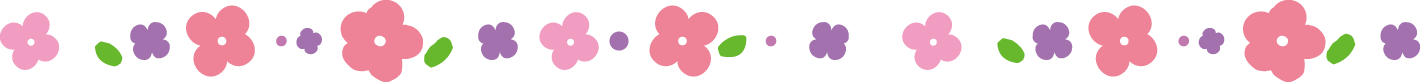


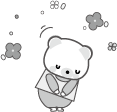
　 　　That's all for the questionnaire.

Thank you very much for your cooperation.
